# Prognostic influence of cyclooxygenase-2 protein and mRNA expression in node-negative breast cancer patients

**DOI:** 10.1186/1471-2407-14-952

**Published:** 2014-12-15

**Authors:** Isabel Sicking, Karlien Rommens, Marco J Battista, Daniel Böhm, Susanne Gebhard, Antje Lebrecht, Cristina Cotarelo, Gerald Hoffmann, Jan G Hengstler, Marcus Schmidt

**Affiliations:** Department of Obstetrics and Gynecology, Johannes Gutenberg University, Mainz, Germany; Institute of Pathology, Johannes Gutenberg University, Mainz, Germany; Leibniz Research Centre for Working Environment and Human Factors (IfADo), Dortmund University of Technology, Dortmund, Germany

**Keywords:** COX-2, Breast cancer, Node-negative, Prognosis

## Abstract

**Background:**

Cyclooxygenases (COX) play a key role in prostaglandin metabolism and are important for tumor development and progression. The aim of this study was to analyze the prognostic impact of COX-2 expression in a cohort of lymph node-negative breast cancer patients not treated in the adjuvant setting.

**Methods:**

COX-2 expression was determined by immunohistochemistry (IHC) in tumor tissue of 193 node-negative breast cancer patients. Additionally, mRNA expression was determined in corresponding tumor samples using microarray based gene-expression data. Univariate and multivariate Cox regression analyses adjusted for age at diagnosis, tumor size, histological grade, human epithelial growth factor receptor 2 (HER2), estrogen receptor (ER) and progesterone receptor (PR) were performed to evaluate the association of both COX-2 protein and mRNA expression with survival. Survival rates were determined by the Kaplan-Meier method. Correlations between COX-2 expression and established prognostic factors were analyzed using the Chi-square test. A potential correlation between COX-2 protein expression and COX-2 mRNA expression was assessed utilizing the Kruscal-Wallis-H-test.

**Results:**

COX-2 protein expression was positive in 24.9% of the breast cancer samples. Univariate analysis showed that COX-2 protein expression was associated with shorter disease-free survival (DFS) (P = 0.0001), metastasis-free survival (MFS) (P = 0.002) as well as breast cancer specific overall survival (OS) (P = 0.043). In multivariate analysis COX-2 expression retained its significance independent of established prognostic factors for shorter DFS (P < 0.001, HR = 2.767, 95% CI = 1.563-4.901) and for inferior MFS (P = 0.002, HR = 2.7, 95% CI = 1.469-5.263) but not for OS (P = 0.096, HR = 1.929, 95% CI = 0.889-4.187). In contrast, COX-2 mRNA expression was not related to survival and failed to show a correlation with protein expression (P = 0.410).

**Conclusions:**

The present findings support the hypothesis that COX-2 protein but not mRNA expression is associated with an unfavorable outcome in node-negative breast cancer.

**Electronic supplementary material:**

The online version of this article (doi:10.1186/1471-2407-14-952) contains supplementary material, which is available to authorized users.

## Background

It is increasingly recognized that the immune system has a large influence on tumorigenesis. Inflammation is able to promote cancer initiation and progression. The causal relationship between chronic inflammation within the local tissue environment and cancer has been in the focus of research in recent years, leading to the concept of cancer-related inflammation as an emerging hallmark of cancer [1]. Cyclooxygenases regulate the synthesis of prostaglandins and play a substantial role in inflammation. There are two isoforms: Cyclooxygenase-1 is expressed in a constitutive manner whereas Cyclooxygenase-2 (COX-2) is induced by growth factors as well as inflammation and is involved in tumor development and progression [2].

COX-2 selective inhibitors reduce tumorigenesis in rat models and the role of Cox-2 as a target of selective Cox-2 inhibitors in treatment and prevention carcinoma is discussed [3]. In a recent large metaanalysis of patients receiving nonsteroidal anti-inflammatory drugs (NSAID), including COX-2 selective COXibs, NSAID use was associated with reduced risk for breast cancer (relative risk [RR] = 0.88, 95% confidence interval [95% CI] = 0.84 to 0.93) [4]. However, other studies failed to confirm a protective impact of NSAID on breast cancer incidence regardless of the molecular subtype [5]. Considering treatment with selective COX-2 inhibitors, celecoxib resulted in a pre-operative randomized phase II trial in an anti-tumor transcriptional response in primary breast cancer with a substantial decrease in Ki-67 positive cells as compared to placebo [6]. Conversely, the addition of celecoxib to exemestane failed to show an increased benefit in a randomized phase II trial as compared to exemestane alone in metastatic breast cancer [7]. Regarding the prognostic role of Cox-2 expression, results are similarly divided. Some of these biomarker studies described an unfavourable prognostic role of COX-2 in early breast cancer [8–11]. However, other studies failed to show an association of COX-2 and prognosis [12–14]. The vast majority of these studies used immunohistochemistry to examine the expression of COX-2 on the protein level. Investigations analyzing mRNA expression of COX-2 rarely considered an association with prognosis [15, 16]. Furthermore, the studies mentioned above used cohorts of breast cancer patients treated with different adjuvant systemic therapies. Because of this it is hardly possible to clarify whether the impact of COX-2 overexpression is purely prognostic in nature or confounded by predictive effects. Therefore, the aim of the present study was to examine COX-2 expression on the protein as well as on the mRNA level in an untreated cohort of lymph node-negative breast cancer patients in the context of other established prognostic factors.

## Methods

### Study population

The initial study cohort consisted of 410 consecutive lymph node-negative breast cancer patients. Of these 410 patients, tumor tissue for Cox-2 immunohistochemistry as well as for mRNA analysis was available in 193 patients. Patients were treated at the Department of Obstetrics and Gynecology, Johannes Gutenberg University Mainz between the years 1986-1999. Adequate follow-up information of all patients was available. All patients were treated by surgical tumor resection, either modified radical mastectomy (n = 70, 36.3%) or breast conserving surgery followed by irradiation (n = 123, 63.7%), and did not receive any systemic therapy in the adjuvant setting. pT stage was collected from the pathology report of the Gynecological Pathology Division. From the breast cancer database [17], information on age at diagnosis, histological grade, estrogen receptor (ER), progesterone receptor (PR) and human epidermal growth factor receptor 2 (HER2) status were obtained (Table 1). The median follow-up time was 11.2 years. We documented death from cancer or unrelated to breast cancer and recurrence of disease, which include metastasis and local relapse. 31 (16%) patients died from breast cancer, 25 (13.8%) patients died from causes unrelated to breast cancer, and 138 (70.1%) patients were alive at the date of last follow-up, 21 (10.8%) patients suffered from locally-recurrent disease and 45 (23.2%) developed distant metastasis. 5 (2.6%) patients developed contralateral breast cancer. The current study was conducted according to the reporting recommendations for tumor marker prognostic studies (REMARK) [18].

**Table 1:** **Clinicopathological characteristics of node negative breast cancer patients from the Mainz cohort with available gene array and COX-2 immunostaining data (n = 193)**

Characteristics	n	%
**Age at diagnosis**		
<50	44	22.8
≥50	149	77.2
**pT stage**		
pT_1_	105	54.4
pT_2_	85	44.0
pT_3_	3	1.6
**Histological grade**		
G I	41	21.2
G II	105	54.4
G III	47	24.4
**Estrogen receptor status**		
Negative	45	23.3
Positive	148	76.7
**Progesterone receptor status**		
Negative	81	42.0
Positive	112	58.0
**Hormone receptor status** ^1^		
Negative	39	20.2
Positive	154	79.8
**HER-2 status**		
Negative	167	86.5
Positive	26	13.5
**Death**		
Of cancer	30	15.5
Unrelated to cancer	25	12.9
Surviving	138	71.5
**Relapse**	57	29.5
Regional	21	10.9
Metastasis	44	22.8
Contralateral	5	2.6
No relapse	136	70.5
**COX-2 intensity score (IS)**		
0	35	18.1
1	45	23.2
2	65	33.7
3	48	24.9
**COX-2 proportion score (PS)**		
0	35	18.1
1	24	12.4
2	42	21.8
3	33	17.1
4	59	30.6
**COX-2 immunostaining score (product of IS and PS)**		
0	35	18.1
1	11	5.7
2	22	11.4
3	16	8.3
4	30	15.5
6	20	10.4
8	23	11.9
9	9	4.7
12	27	14.0
**COX-2 immunostaining status**		
Negative	145	75.1
Positive	48	24.9

^1^The hormone receptor status is positive as soon as one of both, the estrogen or the progesterone receptor status, is positive.

### Immunohistochemistry

Immunostaining was done on 4 μm thick sections according to standard procedures. Briefly, serial sections of formalin-fixed and paraffin-embedded tumour tissue were subsequently deparaffinized using graded alcohol and xylene. Antigen retrieval reactions were performed in a steamer in citrate buffer of pH10 for 30 minutes. 3% H2O2 solution was applied to block endogenous peroxidase at room temperature for 5 minutes. Monoclonal COX-2 antibody (Clone SP21; DCS, Hamburg, Germany) in a dilution of 1:100 was used to incubate with the tissue sections for 30 minutes at room temperature in a humidified chamber, followed by polymeric biotin–free visualization system (Envision™, DAKO Diagnostic Company, Hamburg, Germany) reaction for 30 minutes at room temperature. Then the sections were incubated with 3,3-diaminobenzidine (DAB) in a dilution of 1:50 with substrate buffer for 5 minutes at room temperature and counterstained with Mayer’s haematoxylin solution for 5 minutes. All slides were mounted and then were evaluated under a Leica light microscope (Leica Microsystem Vertrieb Company, Wetzler, Germany) by two of the authors trained in histological and immunohistochemical diagnostics, unaware of the clinical outcome. All series included appropriate positive and negative controls, and all controls gave adequate results.

### Evaluation of COX-2 immunostaining

Since evaluation of COX-2 expression is not yet standardized, the following scoring criteria were applied: (i) intensity score (IS): intensity of staining was scored as 0 (negative), 1 (weak), 2 (moderate), or 3 (strong), (ii) proportion score (PS) percentage of positive cells was scored as 0 (0% positive cells), 1 (1-10% positive cells), 2 (11-50% positive cells), 3 (51-80% positive cells), or 4 (>80% positive cells). To separate tumors with positive COX-2 expression from tumors with negative COX-2 expression, we regarded the COX-2 immunostaining status as positive when staining intensity was scored 3 and as negative in all other cases. Additionally, we investigated the product of IS and PS as COX-2 immunostaining score, ranging from 0-12.

### Gene array data for fresh frozen tissue

Three previously published datasets for untreated node-negative breast cancer patients were used. The large combined group of 788 patients included the Mainz cohort with 200 patients (GSE11121.), 193 of these with corresponding COX-2 IHC [19], the Rotterdam cohort with 286 patients (GSE2034) [20], and the TRANSBIG cohort with 302 patients (GSE6532, GSE7390) [21, 22]. These cohorts comprise available microarray datasets for medically untreated node-negative breast cancer which have used metastasis-free survival (MFS) as an end point.

### Gene expression profiling and data processing

For the Mainz, Rotterdam, and TRANSBIG cohorts, the Affymetrix, Inc. (Santa Clara, California) Human Genome U133A Array set and GeneChip SystemTM were used to quantify the relative transcript abundance in the breast cancer tissues, as previously described [19], and the robust multiarray average (RMA) algorithm was used for normalization. To analyze COX-2 mRNA expression from the gene array data, probe set 204748_at was used in all cohorts. This probe set has been validated in a previous publication, where the influence of estradiol expression on COX-2 RNA levels has been studied [23]. COX-2 expression was additionally analyzed by qRT-PCR using the following primers: forward: 5′-ATCATAAGCAGGGCCAGCT-3′, reverse: 5′-AAGGCGCAGTTTACGCTGTC-3′, resulting in a 101 bp fragment. Similar results were obtained by probeset 204748_at and by qRT-PCR using the above mentioned primers.

### Ethics Statement

The study was approved by the ethical review board of the medical association of Rhineland-Palatinate, Germany. Informed consent has been obtained and all clinical investigation has been conducted according to the principles expressed in the Declaration of Helsinki.

### Statistical analysis

Univariate and multivariate Cox regression analyses were performed with inclusion to evaluate the association between COX-2 expression in breast carcinoma samples and established prognostic factors such as age at diagnosis, tumor size, histological grade of differentiation, HER2 status, ER and PR with survival time. Dichotomization was done as follows: COX-2 immunostaining status in positive versus negative, age at diagnosis in ≤50 years versus > 50 years, tumor size in pT1 (≤2cm) versus pT2 and pT3 (>2 cm), histological grade of differentiation in G I and II versus G III, HER2 status in positive versus negative and ER status in positive (IRS 1-12) versus negative (IRS 0). Survival rates were determined by the Kaplan-Meier method and survival times were compared using the Log-rank test. Breast cancer-specific disease-free survival (DFS) was specified the time between the date of surgery and the date of loco-regional or metastatic recurrence, breast cancer related death or last follow-up. Metastasis-free survival (MFS) was defined as the time between date of surgery and diagnosis of distant metastasis. Breast cancer specific overall survival (OS) was defined as the time between the date of surgery and the date of death. Patients who died of an unknown or unrelated cause were censored at the date of death. Correlations between COX-2 immunostaining status, age at diagnosis, tumor size, histological grade of differentiation, hormone receptor status, HER2 status, ER and PR were assessed using the Chi-square test. A potential correlation between COX-2 protein expression and COX-2 mRNA expression was assessed using the Kruscal-Wallis-H-test (two-sided test). All P values are two sided. Since no correction for multiple testing was performed, all results must be interpreted as explorative. Statistical analyses were performed using the Statistical Package for Social Science (SPSS) (SPSS Inc, version 20, Chicago, IL, USA).

## Results

### Immunohistochemically determined COX-2 expression independently predicts prognosis

**Figure 1:**
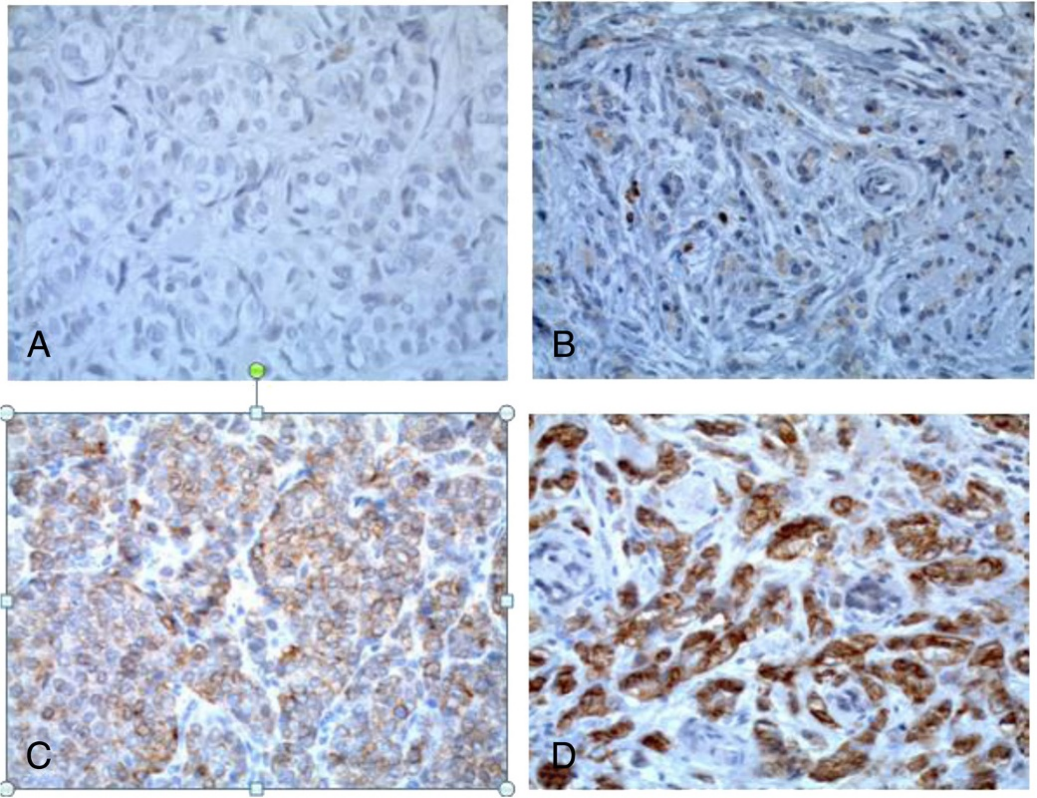
**Representative examples of COX-2 immunohistochemistry in breast carcinoma specimens, A: Staining Intensity (SI) score 0 (absent), B: SI score 1 (weak), C: SI score 2 (moderate), D: SI score 3 (strong); (original magnification: 400-fold).**

To analyze whether COX-2 immunostaining data are associated with prognosis we stained paraffin slices of a cohort of node negative breast carcinomas that recently have been used in Affymetrix RNA profiling studies (Mainz cohort) [19]. Results of immunostaining were assessed using an intensity score (IS: 0-3) and a proportion score (PS: 0-4). Intensity scores of 0-3 were observed for 18.1, 23.2, 33.7 and 24.9% of the patients, respectively (Table 1). Proportion scores of 0-4 were obtained for 18.1, 12.4, 21.8, 17.1 and 30.6%, respectively. Representative pictures of COX-2 immunostaining illustrate that the most striking difference was seen between tumors with the highest possible intensity score of three versus smaller than three (Figure 1). Therefore, we first analyzed DFS in relation to the COX-2 immunostaining status (IS = 3 versus IS < 3). Prognosis of patients with IS = 3 was significantly worse compared to patients with IS < 3 in the univariate (P = 0.001; HR = 2.4) and in the multivariate (P < 0.001; HR = 2.8) Cox analysis, adjusted for age, pTstage, grading, hormone and HER2 status (Table 2). Importantly, the association between COX-2 immunostaining and disease-free survival did not depend on a specific mode of dichotomization of the patients into two groups but all previously reported strategies of immunostaining interpretation resulted in significant results: (i) Intensity and proportion scores were multiplied resulting in an “immunostaining score” (0-12) which was significantly associated with DFS in the multivariate Cox model (P = 0.020; HR = 1.1, Additional file 1: Table S1). (ii) It has been reported that for some prognostic factors only the highest immunostaining score is relevant with respect to prognosis. Therefore, we compared patients with immunostaining scores =12 versus <12 which also led to a significant association with DFS (P = 0.013, HR = 2.4, Additional file 1: Table S2). (iii) Her2 is interpreted as “status positive” when an intensity score of 3 is observed in more than 10% of all tumour cells [24]. Also a “COX-2 status” derived by this rule was significantly associated with DFS (P < 0.001, HR = 3.2, Additional file 1: Table S3). However, it should be considered that “COX-2 status positive” differed from “intensity score = 3” (see above) in only 5 patients which did not have a relevant influence on the result of the Cox analysis. In conclusion, we observed a robust association between immunohistochemically determined COX-2 protein expression and DFS and, hence, used COX-2 immunostaining status as defined by IS = 3 for further analyses.

**Table 2:** **Association of COX-2 immunostaining status (intensity 3** ***vs*** **0-2) with breast cancer specific disease-free survival (DFS) in the Mainz cohort of node negative breast cancer patients (n = 193)**

A. Univariate Cox analysis			
Prognostic factor	p	HR	95% CI
COX-2 immunostaining status	0.001	2.427	1.426-4.131
B. Multivariate Cox analysis			
Prognostic factors	p	HR	95% CI
Age	0.467	0.802	0.443-1.452
(<50 *vs* ≥50 years)			
pT stage	0.815	1.068	0.614-1.861
(≤2cm vs >2cm)			
Histological grade			
(Grade 3 *vs* grade 1 and 2)	<0.001	4.510	2.562-7.940
HR^1^ (ER or PR)	0.523	0.813	0.431-1534
(negative *vs.* positive)			
HER-2 status	0.498	1.273	0.633-2.559
(positive *vs* negative)			
COX-2 immunostaining status	<0.001	2.767	1.563-4.901

^1^The hormone receptor status (HR) is positive as soon as one of both, the estrogen (ER) or the progesterone receptor status (PR), is positive.

### COX-2 and metastasis-free survival

**Figure 2:**
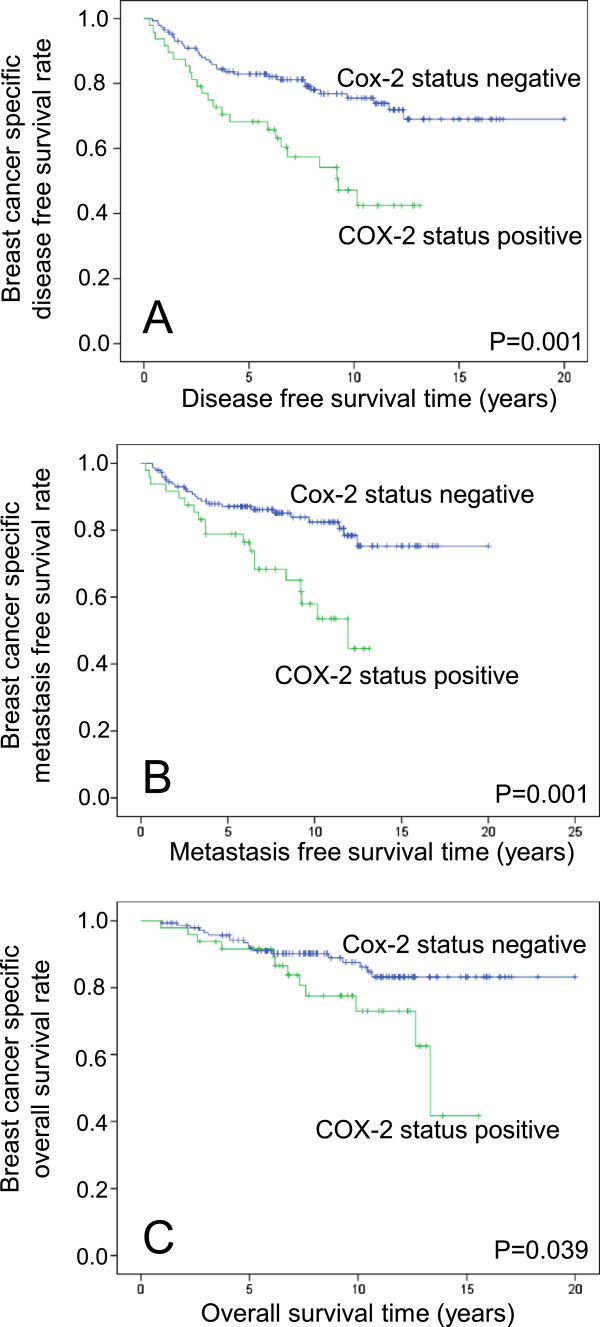
**Positive COX-2 immunostaining status is associated with shorter disease free survival time (A), shorter metastasis free survival time (B) and shorter overall survival (C) time in node-negative breast cancer patients**.

DFS includes the events (i) regional recurrence of breast cancer, (ii) distant metastasis and (iii) contra lateral breast cancer. In a next step we focussed on a possible relationship between COX-2 expression and distant metastasis. Immunohistochemically determined COX-2 was also significantly associated with MFS in the univariate (P = 0.002; HR = 2.6) and multivariate (P = 0.002, HR = 2.7) Cox analysis as shown for the COX-2 intensity score (IS = 3 versus IS < 3) in Table 3. In contrast to DFS and MFS, overall survival (OS) showed only a trend in the multivariate analysis (univariate: P = 0.043, HR = 2.1; multivariate: P = 0.096; HR = 1.929) (Table 4). The worse association for overall survival is not surprising, since (i) the number of death events is smaller compared to relapse events (Table 1) and (ii) several further factors like differences in the treatment of relapsed disease may influence the length of the time period between relapse and death.In a next step DFS and MFS time as well as OS time were visualized by Kaplan-Meier plots. Obviously, the major difference was observed between intensity score 3 and lower scores (Figure 1). Therefore, the dichotomization using the COX-2 immunostaining status (IS = 3 vs <3) seems to be reasonable. In contrast to COX-2 immunostaining status (Figure 2) the COX-2 proportion score (reflecting the fraction of COX-2 positive tumour cells independent from their staining intensity) was not associated with prognosis in Kaplan-Meier analysis (data not shown). This illustrates that identification of patients with high staining intensity is the most critical requirement for immunostaining of COX-2.

**Table 3:** **Association of COX-2 immunostaining status (intensity score 3** ***vs*** **0-2) with breast cancer specific metastasis-free survival (MFS) in the Mainz cohort of node negative breast cancer patients (n = 193)**

A. Univariate Cox analysis			
Prognostic factor	p	HR	95% CI
COX-2 intensity score	0.002	2.582	1.418-4.703
B. Multivariate Cox analysis			
Prognostic factors	p	HR	95% CI
Age	0.693	0.869	0.432-1.747
(<50 *vs* ≥50 years)			
pT stage	0.287	1.411	0.749-2.658
(≤2cm *vs* >2cm)			
Histological grade			
(Grade 3 *vs* grade 1 and 2)	<0.001	4.315	2.275-8.182
HR^1^ (ER or PR)	0.888	0.951	0.471-1.920
(negative *vs.* positive)			
HER-2 status	0.122	1.798	0.855-3.783
(positive *vs* negative)			
COX-2 intensity score	0.002	2.70	1.469-5.263

^1^The hormone receptor status (HR) is positive as soon as one of both, the estrogen (ER) or the progesterone receptor status (PR), is positive.

**Table 4:** **Association of COX-2 immunostaining status (intensity 3 vs 0-2) with breast cancer specific overall survival (OS) in the Mainz cohort of node negative breast cancer patients (n = 193)**

A. Univariate Cox analysis			
Prognostic factor	p	HR	95% CI
COX-2 immunostaining status	0.043	2.128	1.023-4.427
B. Multivariate Cox analysis			
Prognostic factors	p	HR	95% CI
Age	0.984	0.991	0.418-2.350
(<50 *vs* ≥50 years)			
pT stage	0.547	1.262	0.592-2.693
(≤2cm vs >2cm)			
Histological grade			
(Grade 3 *vs* grade 1 and 2)	<0.001	5.331	2.325-12.223
HR^1^ (ER or PR)	0.812	0.903	0.391-2.089
(Negative *vs.* positive)			
HER-2 status	0.290	1.592	0.672-3.770
(Positive *vs* negative)			
COX-2 immunostaining status	0.096	1.929	0.889-4.187

^1^The hormone receptor status (HR) is positive as soon as one of both, the estrogen (ER) or the progesterone receptor status (PR), is positive.

### COX-2 mRNA expression does not correlate with protein levels and is not associated with prognosis

**Figure 3:**
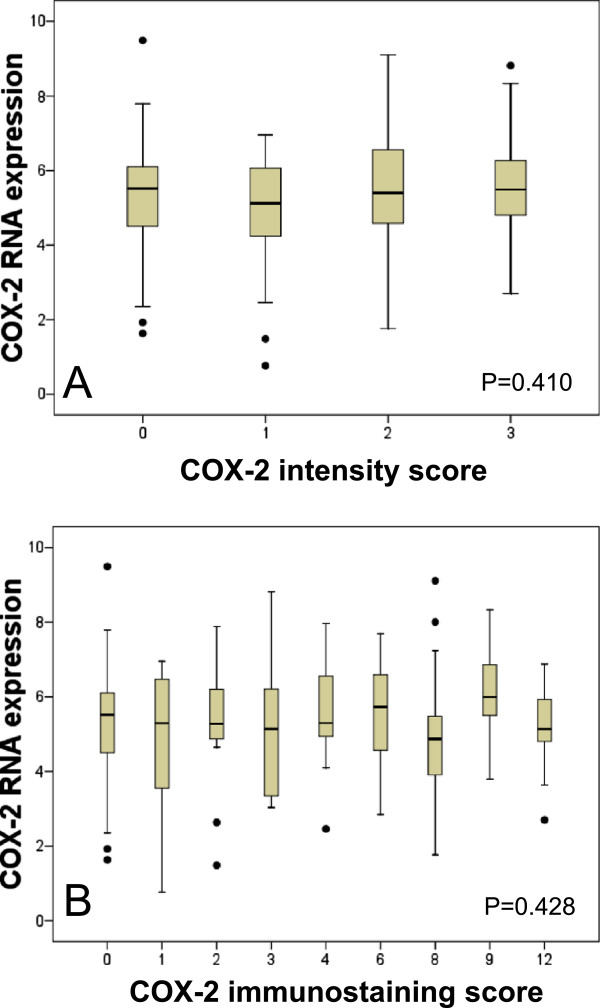
**Correlation of COX-2 mRNA with COX-2 intensity score (A) and COX-2 immunostaining score (B).**

The same tumours from the Mainz cohort that have been studied by immunostaining were analyzed for COX-2 mRNA expression using Affymetrix microarrays [19]. Neither the COX-2 intensity score nor the proportion score correlated with COX-2 mRNA expression (Figure 3). COX-2 mRNA expression was not associated with DFS, MFS and OS, neither in the univariate nor in the multivariate Cox model (Additional file 1: Tables S4-S6). To analyze whether the lack of association between COX-2 mRNA expression and prognosis may be due to a too low case number in our cohort (n = 193), we additionally included two further previously published cohorts of node-negative breast cancer patients into this study, namely the Rotterdam (n = 286) and the TRANSBIG (n = 302) cohorts. In none of these cohorts was high COX-2 RNA expression associated with worse prognosis. Even if we combined all three cohorts leading to a large group of 788 patients with node-negative breast cancer, no association between COX-2 mRNA expression and metastasis free survival was obtained (Additional file 1: Table S7).

### Correlation of COX-2 protein expression with other established prognostic factors

Furthermore, we investigated correlations of the COX-2 immunostaining status with well-established prognostic factors. COX-2 protein expression failed to show an association with age at diagnosis (P = 0.708) (Additional file 1: Table S8A), tumor size (P = 0.508) (Additional file 1: Table S8B), histologic grading (P = 0.904) (Additional file 1: Table S8C), hormone receptor status (P = 0.125) (Additional file 1: Table S8D), PR (P = 0.773) (Additional file 1: Table S8E) or HER2 status (P = 0.453) (Additional file 1: Table S8F). Only ER showed an association with COX-2 protein expression (P = 0.041) (Additional file 1: Table S8G). ER positive carcinomas were more likely to show COX-2 protein expression.

## Discussion

Prognostic markers are needed to define node-negative high-risk patients who would benefit from additional adjuvant systemic treatment. To the best of our knowledge the current study is the first to examine the prognostic impact of COX-2 expression in patients with node-negative breast carcinoma, who received no systemic treatment in the adjuvant setting. This cohort allows the assessment of the pure prognostic effect of COX-2 without any confounding predictive effects. In our study, increased protein expression of COX-2 using a COX-2 immunostaining status (IS = 3) was detected in 22.3% of the breast carcinoma samples. Positive COX-2 protein expression was associated with shorter DFS, MFS, and OS in univariate analysis. COX-2 expression was also correlated to DFS and MFS independent of other established prognostic factors. For OS, this correlation showed only a trend. The worse association for overall survival in our study is not surprising, since the number of death events is smaller compared to relapse events and several further factors may influence the length of the time period between relapse and death.

The expression of COX-2 in breast cancer has been observed in several studies. COX-2 protein expression varies from 17.4% [25] to 57.3% [26]. This diversity of COX-2 positivity in breast cancer may be due to different analytical methods, cut-off values and patient characteristics. In the present study we defined a COX-2 positive status only when staining intensity was scored 3, explaining why the detection rate was comparably low with 22.3%. This is similar to Kim and co-workers who regarded a staining intensity of 2 and 3 as positive [27]. In their study of postmastectomy chest wall relapse, COX-2 protein expression correlated with increased distant metastasis [27]. In line with their findings, several other retrospective studies have reported that increased protein expression of COX-2 is a negative prognostic marker for increased metastasis or reduced overall survival in primary breast cancer [9, 8, 10, 11]. However, the association of COX-2 and survival remains controversial [28–31]. For instance, Holmes and co-workers reported recently that the higher risk of breast cancer death among women with COX-2 positive tumors was fully accounted for by worse stage at diagnosis [28]. However, since the aforementioned studies are retrospective and differ in composition of the examined cohorts of patients as well as in the study design, we felt that further studies in a more homogeneous cohort of breast cancer patients were needed to investigate the impact of COX-2 expression on prognosis.

The association of COX-2 with established prognostic factors is similarly controversial. Contrary to several studies relating COX-2 expression to parameters that characterized the aggressiveness of breast cancer, such as large tumor size, axillary lymph node metastasis, high histologic grading, negative hormone receptor status and positive Her-2 status, [9, 11, 10, 30, 8] our results indicate that COX-2 protein expression has a positive correlation with ER.

The strength of the present study is that we included only patients with node-negative breast cancer not treated in the adjuvant setting, suggesting that in early breast cancer COX-2 expression is indeed independent of other prognostic factors. Moreover, the same tumor tissue specimens analyzed by immunohistochemistry in the present study have also been analyzed by Affymetrix gene arrays. We found that COX-2 mRNA expression does not correlate with protein expression and that, contrary to COX-2 protein expression, mRNA expression is not related to outcome. Boneberg and co-workers compared expression profiles of COX-2 in 48 breast cancer tissues, 41 tumor-adjacent tissues, and 12 breast tissue samples utilizing RT-PCR [32]. Surprisingly, the expression of COX-2 mRNA was decreased in the breast cancer samples not overexpressed as previously reported using immunohistochemistry. A potential association with survival was not examined in their study. Similarly, the study of McCarthy also used real-time RT-PCR in small cohort of breast cancer samples (n = 45) without looking at the prognostic impact of COX-2 mRNA expression [16]. In contrast to our negative results with COX-2 mRNA levels we found a highly significant association between COX-2 immunohistochemistry and outcome in the same cohort of node-negative breast cancer patients. The most likely explanation for these seemingly discrepant results is that COX-2 protein levels in breast cancer tissue predominantly depend on translation and protein stability. Therefore, COX-2 protein measured with immunohistochemistry seems to be more relevant for prognosis than COX-2 mRNA levels.

## Conclusions

In conclusion, our results provide further evidence that increased COX-2 protein expression is associated with poor disease-free survival and metastasis-free survival independent of other prognostic factors. In this context it is tempting to speculate that treatment with a selective COX-2 inhibitor might improve the poor prognosis of patients with overexpression of COX-2. Even though our study presents a well-characterized and homogenous cohort of node-negative breast cancer patients not treated in the adjuvant setting, which takes both potential predictive effects as well as a relationship of COX-2 with increased stage of disease out of the equation, it suffers from the usual limitations of a retrospective study design. Because of this, the proposed prognostic impact of COX-2 expression in early breast cancer has to be interpreted with caution. Prospective studies will be necessary to evaluate the prognostic effect of COX-2 protein expression in breast cancer patients.

## Authors’ information

Marcus Schmidt and Jan. G. Hengstler are shared senior authors.

## Electronic supplementary material

Additional file 1: **Supplementary information.** (DOC 164 KB)
