# Supplementary material for: Prognostic influence of cyclooxygenase-2 protein and mRNA expression in node-negative breast cancer patients
Source: BMC Cancer. 2014 Dec 15;14:952. doi: 10.1186/1471-2407-14-952 (PMC4302078; doi:10.1186/1471-2407-14-952)
Supplement: Supplementary file 1 — Additional file 1: Supplementary information. (DOC 164 KB) [file 12885_2014_5104_MOESM1_ESM.doc]

**Supplementary information**

**Table S1:** Association of COX-2 immunostaining score (1-12) with breast cancer specific **disease-free survival (DFS)** in the Mainz cohort of node negative breast cancer patients (n=193)

A. Univariate Cox analysis

| Prognostic factor | p | HR | 95% CI |
| --- | --- | --- | --- |
| COX-2 immunostaining score (1-12) | 0.078 | 1.058 | 0.994-1.126 |

B. Multivariate Cox analysis

| Prognostic factors | p | HR | 95% CI |
| --- | --- | --- | --- |
| Age  (<50 *vs* ≥50 years) | 0.725 | 0.898 | 0.493-1.636 |
| pT stage  (≤2cm *vs* >2cm) | 0.793 | 1.078 | 0.616-1.887 |
| Histological grade  (Grade 3 *vs* grade  1 and 2) | <0.001 | 5.050 | 2.812-9.069 |
| HR^1^ (ER or PR)  (negative *vs.* positive) | 0.883 | 0.954 | 0.510-1.784 |
| HER-2 status  (positive *vs* negative) | 0.766 | 1.115 | 0.545-2.281 |
| COX-2 immunostaining score (1-12) | 0.020 | 1.089 | 1.014-1.169 |

^1^The hormone receptor status (HR) is positive as soon as one of both, the estrogen (ER) or the progesterone receptor status (PR), is positive.

**Table S2:** Association of COX-2 immunostaining score (12 *vs* <12) with breast cancer specific **disease-free survival (DFS)** in the Mainz cohort of node negative breast cancer patients (n=193)

A. Univariate Cox analysis

| Prognostic factor | p | HR | 95% CI |
| --- | --- | --- | --- |
| COX-2 immunostaining score (12 vs<12) | 0.104 | 1.679 | 0.897-3.211 |

B. Multivariate Cox analysis

| Prognostic factors | p | HR | 95% CI |
| --- | --- | --- | --- |
| Age  (<50 *vs* ≥50 years) | 0.555 | 0.837 | 0.463-1.512 |
| pT stage  (≤2cm *vs* >2cm) | 0.956 | 0.984 | 0.565-1.714 |
| Histological grade  (Grade 3 *vs* grade  1 and 2) | <0.001 | 5.326 | 2.938-9.657 |
| HR^1^ (ER or PR)  (negative *vs.* positive) | 0.856 | 1.071 | 0.513-2.235 |
| HER-2 status  (positive *vs* negative) | 0.856 | 1.071 | 0.513-2.235 |
| COX-2 immunostaining score (12 *vs* <12) | 0.013 | 2.407 | 1.205-4.809 |

^1^The hormone receptor status (HR) is positive as soon as one of both, the estrogen (ER) or the progesterone receptor status (PR), is positive.

**Table S3:** Association of positive COX-2 immunostaining status (intensity score = 3 in at least 10% of all tumor cells *vs* patients with lower expression levels) with breast cancer specific **disease-free survival (DFS)** in the Mainz cohort of node negative breast cancer patients (n=193)

A. Univariate Cox analysis

| Prognostic factor | p | HR | 95% CI |
| --- | --- | --- | --- |
| COX-2 immunostaining status (positive vs negative) | 0.001 | 2.421 | 1.410-4.155 |

B. Multivariate Cox analysis

| Prognostic factors | p | HR | 95% CI |
| --- | --- | --- | --- |
| Age  (<50 *vs* ≥50 years) | 0.701 | 0.889 | 0.489-1.619 |
| pT stage  (≤2cm *vs* >2cm) | 0.774 | 1.085 | 0.622-1.893 |
| Histological grade  (Grade 3 *vs* grade  1 and 2) | <0.001 | 5.211 | 2.913-9.323 |
| HR^1^ (ER or PR)  (negative *vs.* positive) | 0.676 | 0.874 | 0.466-1.642 |
| HER-2 status  (positive *vs* negative) | 0.778 | 1.109 | 0.466-1.642 |
| COX-2 immunostaining status (positive *vs* negative) | <0.001 | 3.190 | 1.778-5.723 |

^1^The hormone receptor status (HR) is positive as soon as one of both, the estrogen (ER) or the progesterone receptor status (PR), is positive.

**Table S4:** Association of COX-2 RNA expression (log2 transformed data) with breast cancer specific **disease-free survival (DFS)** in the Mainz cohort of node negative breast cancer patients (n=193)

A. Univariate Cox analysis

| Prognostic factor | p | HR | 95% CI |
| --- | --- | --- | --- |
| COX-2 RNA expression | 0.485 | 1.067 | 0.890-1.278 |

B. Multivariate Cox analysis

| Prognostic factors | p | HR | 95% CI |
| --- | --- | --- | --- |
| Age  (<50 *vs* ≥50 years) | 0.500 | 0.817 | 0.453-1.471 |
| pT stage  (≤2cm vs >2cm) | 0.826 | 0.941 | 0.545-1.624 |
| Histological grade  (Grade 3 *vs* grade  1 and 2) | <0.001 | 4.920 | 2.785-8.693 |
| HR^1^ (ER or PR)  (negative *vs.* positive) | 0.738 | 1.112 | 0.596-2.073 |
| HER-2 status  (positive *vs* negative) | 0.475 | 1.287 | 0.644-2.572 |
| COX-2 RNA expression | 0.637 | 1.045 | 0.871-1.253 |

^1^The hormone receptor status (HR) is positive as soon as one of both, the estrogen (ER) or the progesterone receptor status (PR), is positive.

**Table S5:** Association of COX-2 RNA expression (log2 transformed data) with breast cancer specific **metastasis-free survival (MFS)** in the Mainz cohort of node negative breast cancer patients (n=193)

A. Univariate Cox analysis

| Prognostic factor | p | HR | 95% CI |
| --- | --- | --- | --- |
| COX-2 RNA expression | 0.570 | 1.059 | 0.868-1.293 |

B. Multivariate Cox analysis

| Prognostic factors | p | HR | 95% CI |
| --- | --- | --- | --- |
| Age  (<50 *vs* ≥50 years) | 0.729 | 0.886 | 0.446-1.759 |
| pT stage  (≤2cm vs >2cm) | 0.672 | 1.142 | 0.618-2.108 |
| Histological grade  (Grade 3 *vs* grade  1 and 2) | <0.001 | 4.913 | 2.599-9.288 |
| HR^1^ (ER or PR)  (negative *vs.* positive) | 0.539 | 1.246 | 0.617-2.517 |
| HER-2 status  (positive *vs* negative) | 0.164 | 1.688 | 0.807-3.531 |
| COX-2 RNA expression | 0.692 | 1.042 | 0.850-1.277 |

^1^The hormone receptor status (HR) is positive as soon as one of both, the estrogen (ER) or the progesterone receptor status (PR), is positive.

**Table S6:** Association of COX-2 RNA expression (log2 transformed data) with breast cancer specific **overall** **survival (OS)** in the Mainz cohort of node negative breast cancer patients (n=193)

A. Univariate Cox analysis

| Prognostic factor | p | HR | 95% CI |
| --- | --- | --- | --- |
| COX-2 RNA expression | 0.390 | 1.109 | 0.876-1.404 |

B. Multivariate Cox analysis

| Prognostic factors | p | HR | 95% CI |
| --- | --- | --- | --- |
| Age  (<50 *vs* ≥50 years) | 0.948 | 0.973 | 0.421-2.247 |
| pT stage  (≤2cm vs >2cm) | 0.818 | 1.091 | 0.519-2.292 |
| Histological grade  (Grade 3 *vs* grade  1 and 2) | <0.001 | 6.358 | 2.810-14.384 |
| HR^1^ (ER or PR)  (negative *vs.* positive) | 0.674 | 1.202 | 0.510-2.831 |
| HER-2 status  (positive *vs* negative) | 0.334 | 1.526 | 0.647-3.597 |
| COX-2 RNA expression | 0.606 | 1.068 | 0.833-1.368 |

^1^The hormone receptor status (HR) is positive as soon as one of both, the estrogen (ER) or the progesterone receptor status (PR), is positive.

**Table S7:** Association of COX-2 RNA expression (log2 transformed data) with breast cancer specific **metastasis-free survival (MFS)** in 788 patients with node-negative breast cancer (combined Mainz, Rotterdam and Transbig) cohorts

Univariate Cox analysis

| Prognostic factor | p | HR | 95% CI |
| --- | --- | --- | --- |
| COX-2 RNA expression | 0.292 | 0.956 | 0.880-1.039 |

**Table S8:** Association of age, pTstage, grading, hormone receptor status, progesterone receptor status, HER2 status and estrogen receptor status with COX-2 immunostating status (CIS=3 versus CIS<3).

**A.** Age is not associated with COX-2 immunostaining status (P=0.708; Chi-square test)

|  | | | | | |
| --- | --- | --- | --- | --- | --- |
|  |  |  | COX-2 immunostaining status | | Total |
|  |  |  | negative | positive |  |
| Age (years) | <50 | number | 34 | 10 | 44 |
|  |  | *%* | *23.4%* | *20.8%* | *22.8%* |
|  | ≥50 | number | 111 | 38 | 149 |
|  |  | *%* | *76.6%* | *79.2%* | *77.2%* |
| Total | | number | 145 | 48 | 193 |
|  |  | *%* | *100.0%* | *100.0%* | *100.0%* |

**B.** pTstage is not associated with COX-2 immunostaining status (P=0.528; Chi-square test)

|  | | | | | |
| --- | --- | --- | --- | --- | --- |
|  |  |  | COX-2 immunostaining status | | Total |
|  |  |  | negative | positive |  |
| pTstage | ≤2cm | number | 77 | 28 | 105 |
|  |  | *%* | *53.1%* | *58.3%* | *54.4%* |
|  | >2cm | number | 68 | 20 | 88 |
|  |  | *%* | *46.9%* | *41.7%* | *45.6%* |
| Total | | number | 145 | 48 | 193 |
|  |  | *%* | *100.0%* | *100.0%* | *100.0%* |

**C.** Histologic grading is not associated with COX-2 immunostaining status (P=0.904; Chi-square test)

|  | | | | | |
| --- | --- | --- | --- | --- | --- |
|  |  |  | COX-2 immunostaining  status | | Total |
|  |  |  | negative | positive |  |
| Grading | G1, G2 | number | 110 | 36 | 146 |
|  |  | *%* | *75.9%* | *75.0%* | *75.6%* |
|  | G3 | number | 35 | 12 | 47 |
|  |  | *%* | *24.1%* | *25.0%* | *24.4%* |
| Total | | number | 145 | 48 | 193 |
|  |  | *%* | *100.0%* | *100.0%* | *100.0%* |

**D.** Hormone receptor status is not associated with COX-2 immunostaining status (P=0.125; Chi-square test). Hormone receptor status is defined as positive if either estrogen or progesterone receptor status is positive.

|  | | | | | |
| --- | --- | --- | --- | --- | --- |
|  |  |  | COX-2 immunostaining  status | | Total |
|  |  |  | negative | positive |  |
| Hormone  receptor  status | negative | number | 33 | 6 | 39 |
|  |  | *%* | *22.8%* | *12.5%* | *20.2%* |
|  | positive | numer | 112 | 42 | 154 |
|  |  | *%* | *77.2%* | *87.5%* | *79.8%* |
| Total | | number | 145 | 48 | 193 |
|  |  | *%* | *100.0%* | *100.0%* | *100.0%* |

**E.** Progesterone receptor status is not associated with COX-2 immunostaining status (P=0.773; Chi-square test).

|  | | | | | |
| --- | --- | --- | --- | --- | --- |
|  |  |  | COX-2 immunostaining  status | | Total |
|  |  |  | negative | positive |  |
| Proges-terone  Receptor  status | negative | number | 60 | 21 | 81 |
|  |  | *%* | *41.4%* | *43.8%* | *42.0%* |
|  | positive | number | 85 | 27 | 112 |
|  |  | *%* | *58.6%* | *56.3%* | *58.0%* |
| Total | | number | 145 | 48 | 193 |
|  |  | *%* | *100.0%* | *100.0%* | *100.0%* |

**F.** HER2 status is not associated with COX-2 immunostaining status (P=0.453; Chi-square test).

|  | | | | | |
| --- | --- | --- | --- | --- | --- |
|  |  |  | COX-2 immunostaining  status | | Total |
|  |  |  | negative | positive |  |
| Her2 status | negative | number | 127 | 40 | 167 |
|  |  | *%* | *87.6%* | *83.3%* | *86.5%* |
|  | positive | number | 18 | 8 | 26 |
|  |  | *%* | *12.4%* | *16.7%* | *13.5%* |
| Total | | number | 145 | 48 | 193 |
|  |  | *%* | *100.0%* | *100.0%* | *100.0%* |

**G.** Estrogen receptor status is associated with COX-2 immunostaining status (P=0.041; Chi-square test).

|  | | | | | |
| --- | --- | --- | --- | --- | --- |
|  |  |  | COX-2 immunostaining  status | | Total |
|  |  |  | negative | positive |  |
| Estrogen  Receptor  status | negative | number | 39 | 6 | 45 |
|  |  | *%* | *26.9%* | *12.5%* | *23.3%* |
|  | positive | number | 106 | 42 | 148 |
|  |  | *%* | *73.1%* | *87.5%* | *76.7%* |
| Total | | number | 145 | 48 | 193 |
|  |  | *%* | *100.0%* | *100.0%* | *100.0%* |
